# Hemin–G-quadruplex-crosslinked poly-*N*-isopropylacrylamide hydrogel: a catalytic matrix for the deposition of conductive polyaniline†
†Electronic supplementary information (ESI) available. See DOI: 10.1039/c5sc02203g


**DOI:** 10.1039/c5sc02203g

**Published:** 2015-08-10

**Authors:** Chun-Hua Lu, Weiwei Guo, Xiu-Juan Qi, Avner Neubauer, Yossi Paltiel, Itamar Willner

**Affiliations:** a Institute of Chemistry and The Center for Nanoscience and Nanotechnology , The Hebrew University of Jerusalem , Jerusalem 91904 , Israel . Email: willnea@vms.huji.ac.il; b The Key Laboratory of Analysis and Detection Technology for Food Safety of the MOE , College of Chemistry , Fuzhou University , Fuzhou 350002 , China; c Applied Physics Department , Faculty of Science , The Hebrew University of Jerusalem , Jerusalem 91904 , Israel

## Abstract

A G-rich nucleic acid-tethered acrylamide/*N*-isopropylacrylamide (NIPAM) copolymer is prepared. The nucleic acid-modified pNIPAM chains assemble, in the presence of K^+^ ions, into a stimuli-responsive G-quadruplex-crosslinked pNIPAM hydrogel undergoing cyclic and reversible solution/hydrogel/solid transitions. Addition of kryptofix [2.2.2], CP, to the K^+^-stabilized G-quadruplex-crosslinked hydrogel eliminates the K^+^ ions from the crosslinking units, resulting in the transition of the hydrogel into a pNIPAM solution. In turn, heating the pNIPAM hydrogel from 25 °C to 40 °C results in the transition of the hydrogel to the solid state, and cooling the solid to 25 °C restores the hydrogel state. Incorporation of hemin into the G-quadruplex-crosslinked hydrogel results in a catalytic hydrogel that catalyzes the oxidation of aniline by H_2_O_2_ to form polyaniline. The polyaniline/pNIPAM hydrogel hybrid doped with 2 M HCl forms an emeraldine salt, which exhibits an electrical conductivity of 9 × 10^–4^ [cm Ω]^–1^.

## Abstract 2

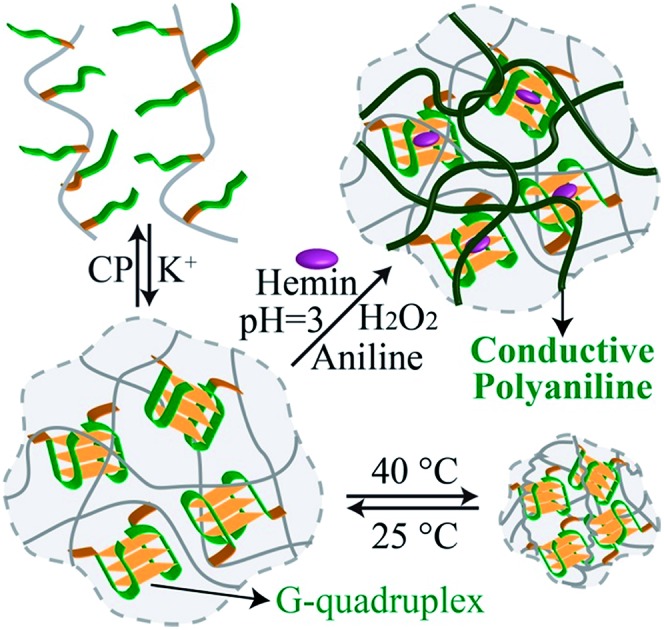
Conducting polyaniline is deposited into a G-quadruplex-crosslinked pNIPAM copolymer undergoing switchable stimuli-responsive solution/hydrogel/solid transitions.

## Introduction

Stimuli-responsive DNA-based hydrogels have attracted recent research efforts.^1^ Two general strategies have been developed to assemble nucleic acid-based hydrogels. One approach utilizes multi-valent DNA branched subunits, which, upon crosslinking with complementary duplex units, yield three-dimensional networks of hydrogels.^2^ The second method involves the tethering of nucleic acid units to polymer chains, *e.g.*, acrylamide, and the crosslinking of the polymer chains by means of nucleic acid tethers.^3^ Stimuli-responsive DNA hydrogels undergoing reversible hydrogel-to-solution phase transitions are tailored by encoding structural information in the DNA crosslinking units that crosslinks or separates the nucleic acid bridging units. Different external triggers, such as strand displacement, metal ion/ligand (*e.g.*, Ag^+^/cysteamine),^4^ pH,^5^ K^+^-stabilized G-quadruplexes/crown ether^6^ or photoisomerization of photoactive units^7^ (*e.g.*, *trans*/*cis* photoisomerization of azobenzene derivatives), have been used to stimulate reversible hydrogel-to-solution phase transitions. Different applications of stimuli-responsive DNA-based hydrogels have been suggested, including controlled drug release,^8^ the development of sensors and biosensors,^9^ the assembly of matrices for the removal of hazardous ions,^10^ the design of shape-memory hydrogels,^11^ and the use of DNAzyme-crosslinked hydrogels for the controlled release of enzymes and the activation of enzyme cascades.^12^

Temperature-responsive hydrogels represent a broad class of macromolecules that undergo reversible solution-to-hydrogel or hydrogel-to-solid temperature-controlled transitions. The most extensively studied thermosensitive polymer is the covalently crosslinked poly-*N*-isopropylacrylamide, pNIPAM, which undergoes a reversible gel-to-solid transition at 32 °C.^13^ The incorporation of metal ions (*e.g.*, Cu^2+^, Hg^2+^, Ag^+^) into the crosslinked hydrogel or the tethering of photoisomerizable groups onto the polymer have been reported to affect the gel-to-solid transition temperature of the crosslinked polymer.^14^ Recently, we reported a pH-responsive DNA-crosslinked pNIPAM hydrogel.^15^ It was demonstrated that pNIPAM chains functionalized with cytosine-rich tethers undergo hydrogelation at pH = 5.2 *via* the crosslinking of the chains by i-motif nanostructures, and that the resulting hydrogel undergoes gel-to-solid transition at 33 °C. Also, it was shown that the i-motif-crosslinked pNIPAM hydrogel dissociates at pH = 7.2 to the solution phase, due to the separation of the i-motif bridging units. In contrast to other crosslinked pNIPAM systems undergoing only thermally induced gel-to-solid transitions, the i-motif-crosslinked pNIPAM shows dual-stimuli responsiveness, undergoing cyclic transitions between solution/hydrogel states (with pH changes) and hydrogel/solid transitions (with temperature changes).

In the present study, we report on the preparation of a reversible dual-stimuli-responsive pNIPAM hydrogel triggered by G-quadruplexes and thermal stimuli, resulting in cyclic transitions between solution–hydrogel–solid states. The association of hemin to the G-quadruplex-crosslinked pNIPAM hydrogel yields a catalytic matrix that catalyzes the oxidation of aniline to polyaniline and the formation of a polyaniline–pNIPAM hydrogel hybrid. Doping of the resulting polyaniline yields a conductive matrix. The use of hemin–G-quadruplex-crosslinked pNIPAM as a catalytic hydrogel for the deposition of conductive polyaniline is innovative, and the results illustrate a new concept for the electronic application of conducting hydrogels. More specifically, simple wet printing or coating processes can be used to manufacture conducting polymers for electronic circuitry.

## Results and discussion

**Fig. 1:**
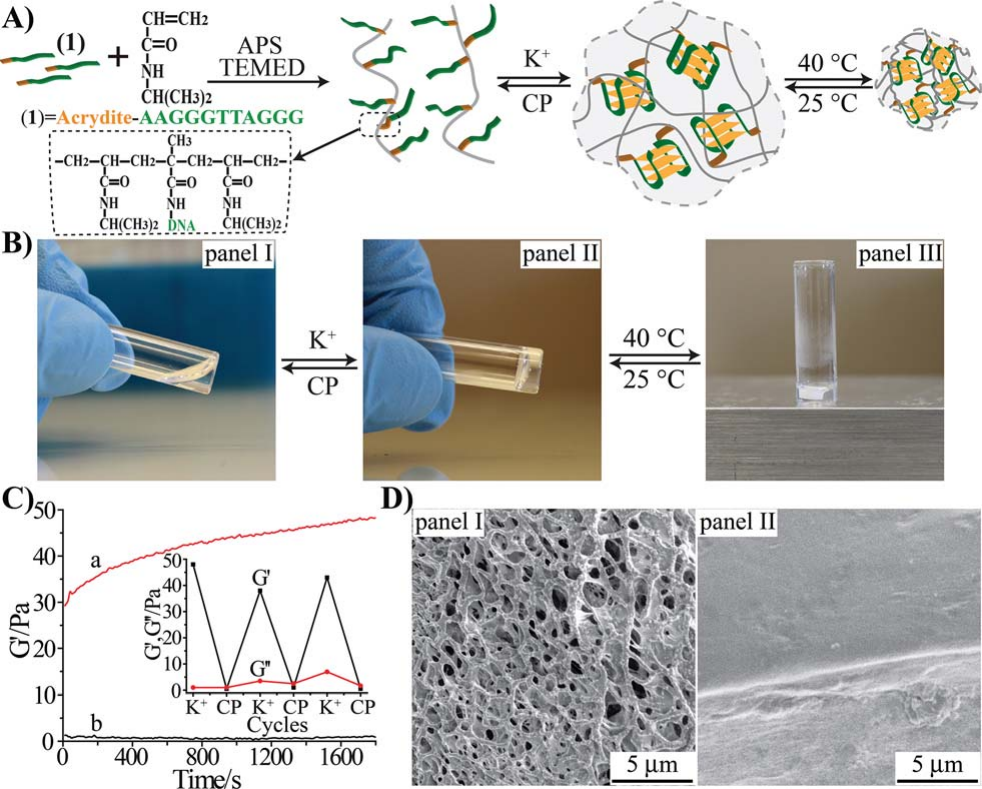
(A) Schematic synthesis of the thermosensitive G-quadruplex-crosslinked (**1**)-acrydite/pNIPAM copolymer switchable hydrogel, which undergoes reversible and cyclic transitions across solution–hydrogel–solid states. (B) Photographic images of the (**1**)-acrydite/pNIPAM copolymer chains in the solution phase, panel I; K^+^-stimulated G-quadruplex-crosslinked hydrogel, panel II; and the solid copolymer phase generated upon heating to 40 °C, panel III. The reversible transitions between the states are also indicated. (C) Time-dependent changes of the storage modulus, *G*′, corresponding to (a) the G-quadruplex-crosslinked (**1**)-acrydite/pNIPAM hydrogel, and (b) the solution consisting of (**1**)-acrydite/pNIPAM copolymer chains. Inset: cyclic and switchable transitions between the liquid solution of the (**1**)-acrydite/pNIPAM polymer chains and the K^+^-stabilized G-quadruplex-crosslinked hydrogel, monitored by following the storage modulus (*G*′) and loss modulus (*G*′′) of the respective phases. The liquid phase of the polymer solution is generated by the dissociation of the G-quadruplex-crosslinked hydrogel in the presence of kryptofix [2.2.2]. (D) Freeze-dried SEM images of the G-quadruplex-crosslinked (**1**)-acrydite/pNIPAM hydrogel, panel I, and of the heated (40 °C) solidified copolymer, panel II.

The preparation of the stimuli-responsive pNIPAM hydrogel is depicted in Fig. 1(A). The acrydite-modified nucleic acid (**1**) was polymerized with *N*-isopropylacrylamide (NIPAM) monomers to yield nucleic acid-functionalized pNIPAM chains. The nucleic acid tethers (**1**) are composed of the half-subunit of the G-quadruplex. The ratio of the NIPAM monomers to **1** in the copolymer chain was 168 : 1 (for the evaluation of the loading see Fig. S1, ESI†). In the presence of K^+^ ions, the inter-chain assembly of G-quadruplexes bridges the pNIPAM chains, resulting in the crosslinked pNIPAM hydrogel, Fig. 1(B), panel II. Treatment of the hydrogel with kryptofix [2.2.2] eliminates the K^+^-ions from the G-quadruplex bridges, resulting in the separation of the hydrogel and the formation of the pNIPAM solution. By the cyclic treatment of the system with K^+^ and kryptofix [2.2.2], the switchable hydrogel-solution transitions of the pNIPAM copolymer proceed. Subjecting the hydrogel to thermal stimulus (40 °C) induces the hydrogel-to-solid transition (Fig. 1(B), panel III), and the reverse cooling of the solid polymer chains (25 °C) restores the hydrogel state. Accordingly, the pNIPAM copolymer undergoes reversible solution–hydrogel–solid transitions in the presence of K^+^/kryptofix [2.2.2] and thermal stimuli, Fig. 1(B). The rheometry studies characterizing the hydrogel system are depicted in Fig. 1(C). The storage modulus, *G*′, of the G-quadruplex-crosslinked pNIPAM hydrogel is *G*′ ≈ 45 Pa, Fig. 1(C), curve (a), which is consistent with the formation of a hydrogel. The liquid phase of the pNIPAM copolymer chains reveals a storage modulus of *G*′ ≈ 1 Pa, Fig. 1(C), curve (b), consistent with the transition of the hydrogel into the liquid phase. By the cyclic treatment of the pNIPAM copolymer system with K^+^ ions and kryptofix [2.2.2], the system is switched between high *G*′ values of 40–50 Pa and *G*′ ≈ 0 Pa, Fig. 1(C), inset. Fig. 1(D) shows the SEM images of the G-quadruplex-crosslinked pNIPAM hydrogel, panel I, and of the thermally solidified polymer matrix, panel II. Evidently, only the hydrogel shows a porous structure.

**Fig. 2:**
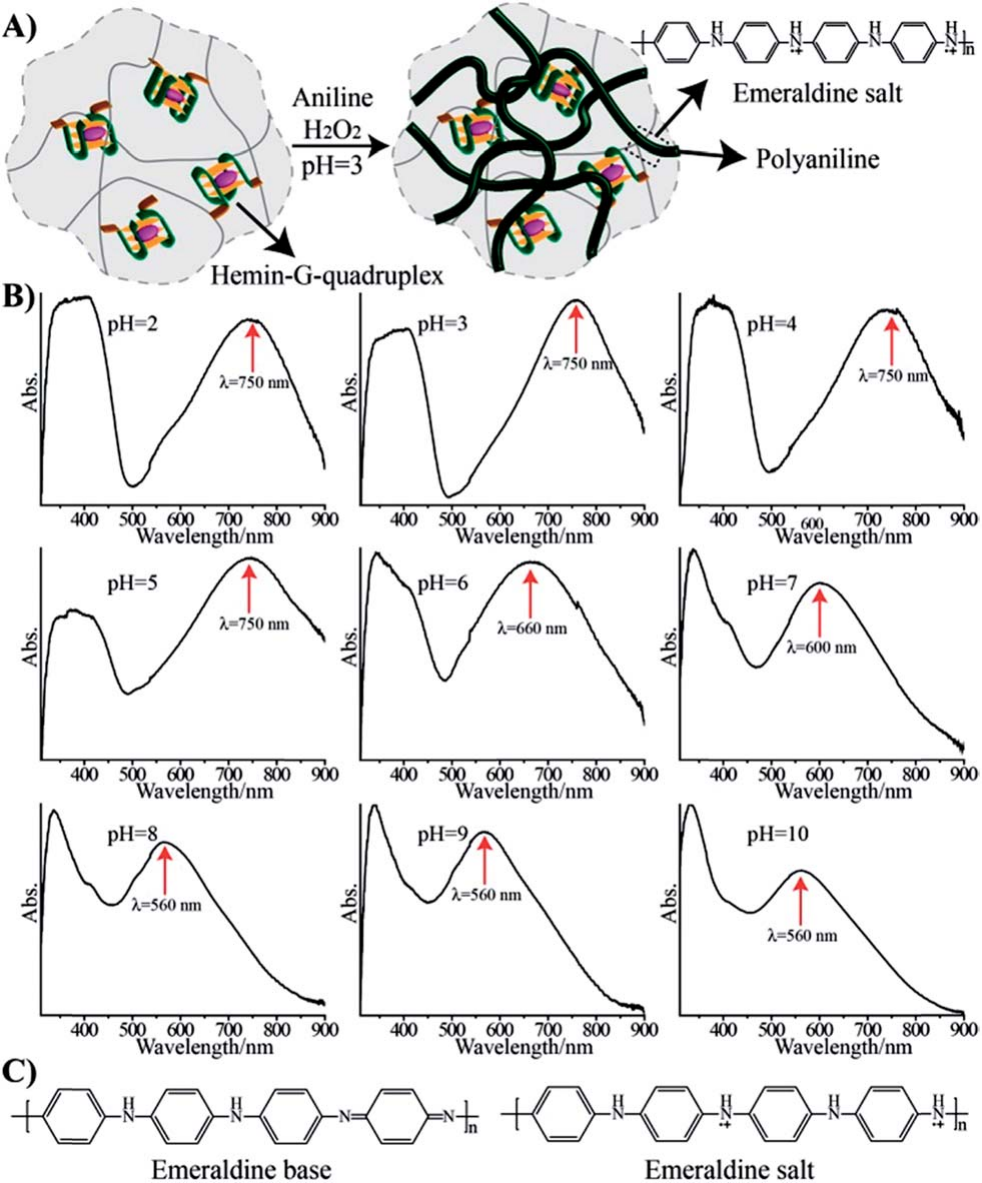
(A) Scheme of the generation of the hemin–G-quadruplex-crosslinked (**1**)-acrydite/pNIPAM hydrogel/polyaniline hybrid through the hemin–G-quadruplex-catalyzed oxidation of aniline by H_2_O_2_ to form polyaniline. (B) Absorption spectra of the hemin–G-quadruplex-crosslinked (**1**)-acrydite/pNIPAM hydrogel/polyaniline hybrid upon subjecting the matrix to different pH environments. (C) Chemical transition between the emeraldine base state of polyaniline and the conducting emeraldine salt doped polyaniline state.

Hemin binds to K^+^-ion-stabilized G-quadruplexes, and the resulting nanostructures reveal horseradish peroxidase (HRP)-mimicking functions.^16^ Specifically, it was demonstrated that hemin–G-quadruplex catalyzed the oxidation of aniline by H_2_O_2_ to polyaniline, which was deposited on nucleic acid scaffolds.^17^ This suggested that a hemin–G-quadruplex-crosslinked pNIPAM hydrogel could act as a catalytic matrix for the deposition of polyaniline onto the hydrogel matrix. Thus, the hemin–G-quadruplex-crosslinked pNIPAM hydrogel might act as a scaffold for the formation of a conductive polymer material. In fact, previous reports have addressed the synthesis of conducting polymers.^18^ Treatment of the hydrogel matrix with aniline and H_2_O_2_ at pH = 3 resulted in a dark green hydrogel, corresponding to the integration of polyaniline into the hydrogel, Fig. 2(A). Subjecting the resulting polyaniline-functionalized hydrogel to different pH environments yielded the absorption spectra shown in Fig. 2(B). A band at 560 nm at pH = 10 is red-shifted to 600 nm and 660 nm at pH = 7 and pH = 6, respectively, and stabilizes to a green hydrogel exhibiting an absorbance band at *λ* = 750 nm at pH = 5. This absorbance band is unchanged even upon lowering the pH of the hydrogel. These spectral changes are consistent with the existence of the polyaniline matrix in the emeraldine base form in the pH-region 6–10, and its transition at pH = 5 and lower, to the emeraldine salt doped conducting form, Fig. 2(C).^19^ The spectral changes associated with the pH-stimulated doping and undoping of the pNIPAM/polyaniline matrix are fully reversible. Rheometry experiments indicate that the resulting hydrogel reveals a substantially higher storage modulus, *G*′ = 85 Pa, as compared to the unmodified hydrogel, *G*′ ≈ 45 Pa (Fig. S2, ESI†). This implies that the resulting polyaniline-functionalized hydrogel is rigidified as compared to the transparent non-modified hydrogel. We note that the storage moduli of the doped and undoped pNIPAM/polyaniline composites exhibit very similar values of 85 ± 6 Pa (Fig. S3†). Also, the pNIPAM/polyaniline hybrid matrix no longer undergoes thermally induced gel-to-solid transitions, and the heating of the pNIPAM/polyaniline hybrid to 40 °C results in only a 30% volume decrease (as compared to a volume change of *ca.* 67% for the thermally stimulated gel-to-solid transition of the transparent G-quadruplex-crosslinked pNIPAM hydrogel). These results suggest that the polyaniline chains entangled within the pNIPAM matrix exist as a non-compressible polymer matrix. Also, cross-section analysis of the pNIPAM/polyaniline composite reveals that the color of polyaniline is distributed throughout the hydrogel matrix, thus indicating that the polyaniline is incorporated through the entire volume of the hydrogel, and that it is not acting as a coating. Further evidence that the polyaniline is entangled in the entire hydrogel matrix was obtained by SEM imaging of the cross-section of the composite material, Fig. S4.† The SEM image of the G-quadruplex-crosslinked polymer hydrogel reveals a porous structure prior to the deposition of polyaniline, *cf.*Fig. 1(D), panel I. After the deposition of polyaniline onto the hemin–G-quadruplex-crosslinked hydrogel, the SEM image of the resulting surface reveals a smooth non-porous surface, Fig. S4,† panel I, consistent with the formation of a rigid, non-elastic matrix. The SEM image of the cross-section of the hemin–G-quadruplex-crosslinked hydrogel, Fig. S4,† panel II, reveals a similar smooth non-porous area, implying that the entire pNIPAM/polyaniline composite exists as a rigidified, non-elastic material, with the polyaniline entangled throughout the composite matrix. Furthermore, it should be noted that the addition of kryptofix [2.2.2] to the solidified polyaniline-functionalized K^+^-stabilized G-quadruplex-crosslinked pNIPAM matrix (40 °C) does not affect the material structure within a time-interval of five hours. Presumably, the solid composite material is non-permeable to the kryptofix ligand, and thus, the G-quadruplex crosslinking units are not dissociated. In turn, addition of kryptofix [2.2.2] to the polyaniline-modified K^+^-stabilized G-quadruplex-crosslinked pNIPAM hydrogel (25 °C) results in the slow fragmentation of the material (*ca.* five hours) into small dark-colored pieces of polyaniline. This suggests that the K^+^-stabilized G-quadruplex crosslinking units of pNIPAM are separated upon the addition of the kryptofix ligand, resulting in the diffusional leakage of the separated pNIPAM chains from the pNIPAM/polyaniline composite, and the formation of polyaniline fragments.

**Fig. 3:**
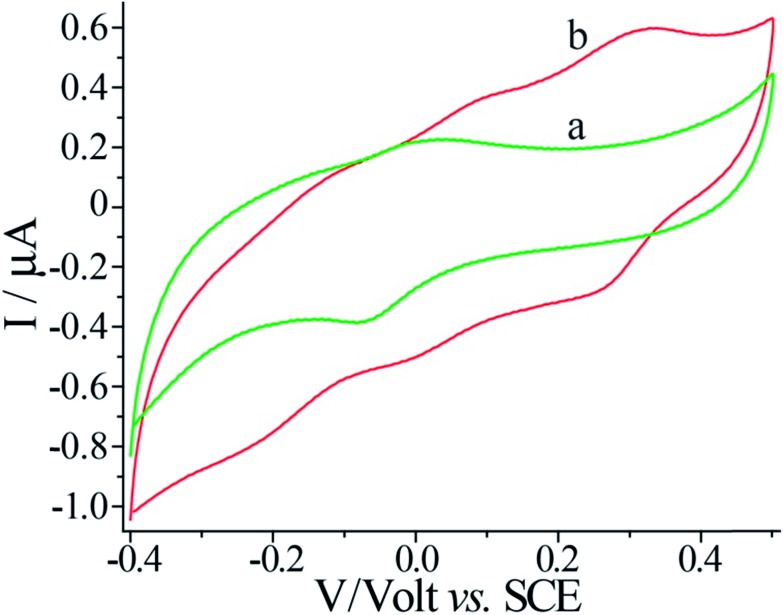
Cyclic voltammograms corresponding to (a) the undoped polyaniline hydrogel at pH = 8 and (b) the proton-doped polyaniline hydrogel (prepared by acidification of the HEPES electrolyte solution with HCl, pH = 3). The polyaniline hydrogel was deposited onto a glassy carbon electrode. Voltammograms were recorded at a scan rate of 100 mV s^–1^; SCE was used as the reference electrode.

**Fig. 4:**
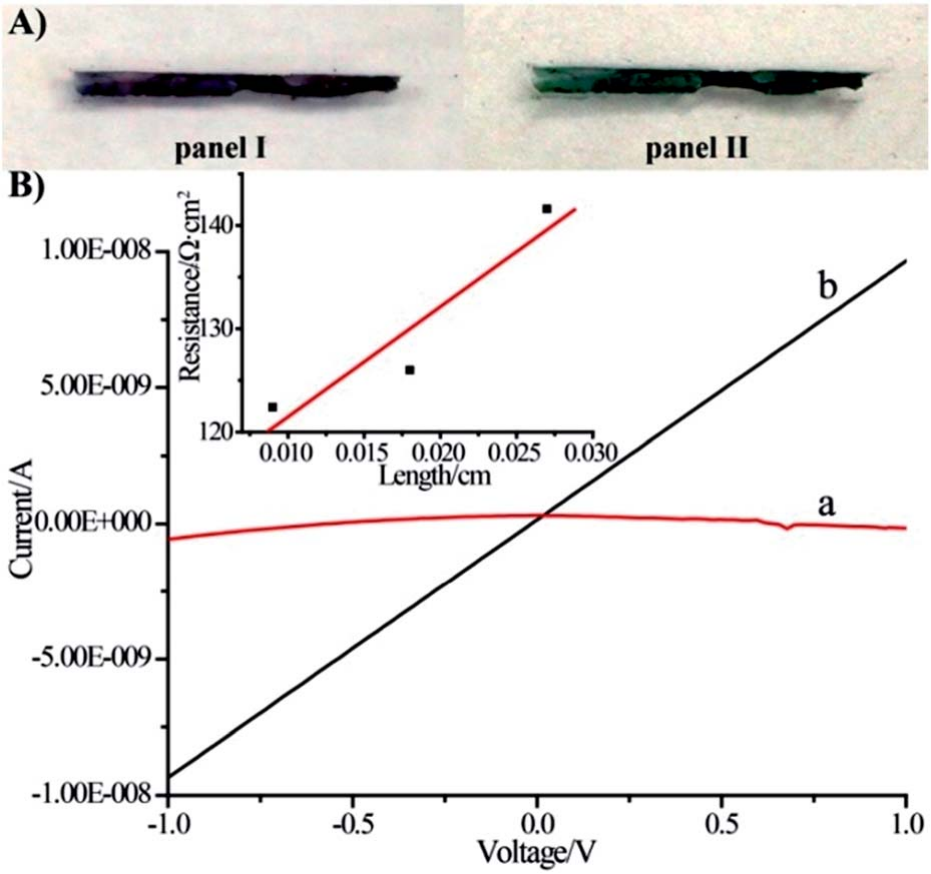
(A) Photographic images of the polyaniline (emeraldine base)/hemin–G-quadruplex-crosslinked (**1**)-acrydite/pNIPAM hydrogel wire, panel I, and of the polyaniline (emeraldine salt)/hemin–G-quadruplex-crosslinked (**1**)-acrydite/pNIPAM hydrogel wire, panel II. (B) *I*–*V* curves corresponding to (a) the wire shown in panel I and (b) the wire shown in panel II. Inset: resistance values between two probe contacts separated by variable distances on the wire shown in panel II.

The voltammetric features of the proton-doped and undoped pNIPAM/polyaniline hydrogel were examined on a glassy carbon electrode, Fig. 3. The pNIPAM/polyaniline hydrogel reveals a quasi-reversible wave at pH = 8, curve (a), at *ca.* –0.05 V *vs.* SCE, consistent with an undoped configuration of polyaniline. At pH = 3, a cyclic voltammogram characteristic of emeraldine proton-doped polyaniline is observed, curve (b).^20^ The emeraldine proton-doped polyaniline salt is recognized as the conducting polyaniline state.^21^ Accordingly, hemin–G-quadruplex-crosslinked pNIPAM hydrogel wires (length 10 mm, cross-section *ca.* 1 mm × 0.1 mm) were prepared in a mold. The hemin–G-quadruplex-crosslinked pNIPAM hydrogels were subjected to the growth of polyaniline for 20 min in the presence of aniline/H_2_O_2_, pH = 3. The resulting polyaniline-functionalized hydrogels were then subjected to pH = 7 to form a purple-colored polyaniline/pNIPAM hydrogel, Fig. 4(A), panel I, or treated with 2 M HCl to yield a dark green-colored, conductive polyaniline/pNIPAM matrix, Fig. 4(A), panel II. The conductivities of the different states of polyaniline/pNIPAM were then evaluated using a probe station. Fig. 4(B), curve (a) depicts the *I*–*V* curve characteristic of the undoped polymer matrix. Evidently, the matrix shows very high resistance, characteristic of an insulating material. In turn, the proton-doped (2 M HCl) polyaniline/pNIPAM wire shows the *I*–*V* curve depicted in Fig. 4(B), curve (b), and from the slope of the curve, the resistance of the matrix was evaluated. The resistances of the wire as a function of length were determined by moving one probe and measuring the *I*–*V* curves. Using a linear fit, we were able to separate the probe contact resistance and the resistance of the wires. The conductivity of the wire (1/resistivity) was then evaluated by plotting the resistance multiplied by the cross-section area as a function of the wire length, Fig. 4(B), inset. The conductivity corresponds to *ca.* 9 × 10^–4^ [cm Ω]^–1^. This value for the conductivity of the polyaniline/pNIPAM wire is in the range of conductivities reported for polyaniline synthesized by other methods, Table 1. The results certainly demonstrate that the proton-doped hemin–G-quadruplex polyaniline/pNIPAM hybrid hydrogel reveals electrical conductivity. The conductivity of the matrix is, however, non-optimized, and improvements in the electrical properties of the polymer may be achieved by tuning the NIPAM/G-quadruplex crosslinking ratio, the time allowed for the DNAzyme-catalyzed deposition of the polyaniline, and other factors. Furthermore, our original vision that the thermally induced gel-to-solid transition of the matrix could establish a thermo-responsive conductivity switch was not realised. We found that the conductivities of the wire at 25 °C and 40 °C are identical (within the experimental error). This observation is consistent with the fact that we could identify only a small volume change upon heating the polyaniline/pNIPAM system.

**Table 1:** Electrical conductivities of conductive hydrogels

Conductive hydrogels^*a*^	Conductivity [cm Ω]^–1^	Ref.
PANI/pNIPAM	2 × 10^–3^	22
PANI/PAMPS	1.3 × 10^–3^	23
PANI/PAC	2.33 × 10^–4^	24
PANI/PAM	0.62	25
PANI/PVA	97.45 × 10^–6^ to 130.3 × 10^–6^	26
G-quadruplex/PANI/pNIPAM	9 × 10^–4^	Present work

^*a*^PANI: polyaniline; PAMPS: poly(2-acrylamido-2-methyl propane sulphonic acid); PAC: polyacrylate; PAM: polyacrylamide; PVA: poly(vinyl alcohol).

## Conclusions

In conclusion, the present paper introduces a thermosensitive G-quadruplex hydrogel that undergoes reversible transitions between solution–hydrogel–solid states. The incorporation of hemin in the crosslinking G-quadruplex units resulted in a catalytic hemin–G-quadruplex, DNAzyme, hydrogel. The catalytic oxidation of aniline by H_2_O_2_ led to the synthesis of a rigidified hydrogel modified with polyaniline. Proton doping of the polyaniline/pNIPAM hybrid resulted in the formation of an electrically conducting matrix. Further challenges to implement the results include the optimization of the conducting properties of the polyaniline/pNIPAM matrices, and the patterning of surfaces with the hemin–G-quadruplex hydrogel to yield electrically conducting circuits.

## Experimental section

### Materials

4-(2-Hydroxyethyl)-1-piperazineethanesulfonic acid (HEPES), ammonium persulfate (APS), *N*,*N*,*N*′,*N*′-tetramethyl-ethylenediamine (TEMED), acrylamide solution (40%), hemin, potassium chloride, kryptofix [2.2.2], aniline and hydrogen peroxide were purchased from Sigma-Aldrich. Desalted 5′ end acrydite-modified nucleic acid strand (**1**) (acrydite-AAGGGTTAGGG) was purchased from Integrated DNA Technologies Inc. (Coralville, IA). Ultrapure water purified by a NANOpure Diamond instrument (Barnstead International, Dubuque, IA, USA) was used to prepare all of the solutions.

### Synthesis of the acrydite nucleic acid (**1**)/pNIPAM copolymer

A buffer solution (HEPES–HCl, 10 mM, pH = 8.0), 200 μL, that included 1.6 mM acrydite nucleic acid (**1**) and 3% NIPAM, was prepared. Nitrogen was bubbled through the solution. Subsequently, 10 μL of a 0.5 mL aqueous solution containing 50 mg APS and 25 μL TEMED was added to the monomer mixture. The resulting mixture was allowed to polymerize at room temperature for five minutes, and then the mixture was further polymerized at 4 °C for 12 hours. The resulting polymer was purified from unreacted monomer units, salts and the initiator using a Microcon (Millipore) spin filter unit (MWCO 10 kD). The purified polymer was removed from the filter and dried under gentle N_2_ flow. The ratio of NIPAM : acrydite nucleic acid was determined spectroscopically.

### Preparation of the G-quadruplex-crosslinked pNIPAM hydrogel and its switchable transitions between hydrogel–solution and hydrogel–solid states

The pNIPAM/acrydite nucleic acid (**1**) copolymer was dissolved in a buffer solution (10 mM HEPES–HCl buffer, pH = 7.0) to yield a copolymer solution containing 1.0 mM nucleic acid (**1**). Potassium chloride, 200 mM, was added to the copolymer mixture to induce gelation by the formation of the G-quadruplex crosslinker units. The gelation was completed within 10 minutes. For the dissociation of the hydrogel, a minute volume of concentrated kryptofix [2.2.2] solution, yielding a concentration of 200 mM in the gel, was added. The dissociation of the hydrogel occurred within 10 minutes.

The G-quadruplex-crosslinked pNIPAM hydrogel was heated to 40 °C to induce gel–solid transitions. The resulting solid was further transformed to the hydrogel state by cooling the copolymer mixtures to room temperature (25 °C).

### Formation of hemin–G-quadruplex polyaniline/pNIPAM hydrogel hybrid

The G-quadruplex-crosslinked pNIPAM hydrogel was immersed in a solution of hemin (50 μM) and Triton 100 (0.05%) for 2 h. After forming the hemin–G-quadruplex, the hydrogel was then transferred into a 50 mM aniline solution (10 mM HEPES–HCl buffer, pH = 3.0) for 1 h. H_2_O_2_ (final concentration 50 mM) was added to the aniline/hydrogel system to initiate the aniline polymerization for 20 min.

## Supplementary Material

Supplementary information
