# Supplementary material for: Hemin–G-quadruplex-crosslinked poly-N-isopropylacrylamide hydrogel: a catalytic matrix for the deposition of conductive polyaniline
Source: Chem Sci. 2015 Aug 10;6(11):6659–64. doi: 10.1039/c5sc02203g (PMC5802269; doi:10.1039/c5sc02203g)
Supplement: Supplementary file 1 [file SC-006-C5SC02203G-s001.pdf]

Electronic Supplementary Material (ESI) for Chemical Science.
This journal is © The Royal Society of Chemistry 2015

Supporting Information

Hemin-G-Quadruplex-Crosslinked Poly-N-Isopropylacrylamide Hydrogels: A Catalytic Matrix for the Deposition of Conductive Polyaniline

Chun-Hua Lu,[a,†] *Weiwei Guo,*[a,†] *Xiu-Juan Qi,*[a,b] *Avner Neubauer,*[c] *Yossi Paltiel,*[c] *and Itamar Willner*[a]*

† These authors contributed equally to this work.

[a] The Institute of Chemistry, The Center for Nanotechnology, The Hebrew University of Jerusalem, Jerusalem, 91904, Israel

[b] The Key Laboratory of Analysis and Detection Technology for Food Safety of the MOE, College of Chemistry, Fuzhou University, Fuzhou 350002, China

[c] Applied Physics Department, Faculty of Science, The Hebrew University, Jerusalem 91904, Israel

E-mail: willnea@vms.huji.ac.il

Experimental Section

Determination of the ratio of NIPAM/acrydite-nucleic acid units in the copolymer chains:

To a solution containing the acrydite nucleic acid (**1**), 2 µM, variable concentrations of the pure pNIPAM polymer were added, and the absorption spectra of the different solutions were recorded. The increase in the absorbance at λ = 200 nm corresponded to the non-substituted pNIPAM chains, while the absorbance at λ = 255 nm corresponded to the acrydite-nucleic acid (**1**). An appropriate calibration curve corresponding to the molar ratio of the nucleic acids in the copolymer and the NIPAM monomer units was derived. Based on this calibration curve, the ratio of NIPAM/acrydite-nucleic acid in the copolymers was evaluated spectroscopically (Figure S1).

Conductivity measurements

The electrical measurements were performed using a Signaotone closed probe station system. The system is mounted on a grounded floating table and is supplying pA current resolution in the IV measurements. The wire like samples were connected from each side by a single probe. Taking different distances between probes, we were able to measure the I-V thus allowing to measure the sample resistance as a function of conductance squares. The resistivity was calculated by taking the slope of the resistance multiplied by the wire cross section area as function of the wire length. The conductivity corresponds to 1/resistivity of the matrix.

Rheology

The in-situ hydrogel formation, mechanical properties, and cross-linking kinetics were characterized by a HAAKE MARS III rheometer (Thermo Scientific). Time-sweep oscillatory tests were performed with a 20 mm parallel-plate geometry, using 120 µL of a pNIPAM copolymer chains solution (resulting in a gap size of 0.26 mm), 1 minute after its preparation, at temperature of 25 °C. In order to find the linear viscoelastic region of the time sweeps, oscillatory strain (0.01–100%) and frequency

sweeps (0.01–100 Hz) were conducted 20 minutes after adding potassium ion to the solution. The linear viscoelastic region was found to be in the range of 1% strain and 1 Hz frequency.

Microscopy

SEM images were taken with Extra High Resolution Scanning Electron Microscope Magellan (TM) 400L, microscope Settings: 2 kV, 6.3 pA. Slides (Si) were first washed with distilled water followed by ethanol and acetone, then UV/ozone cleaned using a T1O×10/OES/E UV/ozone chamber from UVOCS, Inc. (USA). Subsequently, the slides were incubated in 2% aminopropyltriethoxysilane for 30 minutes, and heated to 110˚C for 10 minutes to generate an amino monolayer. A piece of freshly formed hydrogel was placed on the slide surface. Then the hydrogel sample was frozen by immersing it in liquid nitrogen. The frozen hydrogel sample was dried by sublimation of the formed ice under high vacuum, and the resulting surface was further metal-coated with Au/Pd.

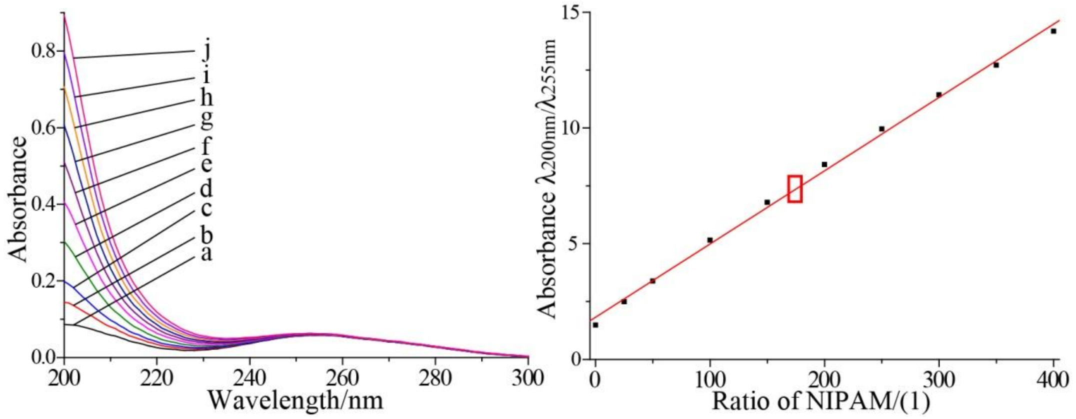

Figure S1. Determination of the loading of pNIPAM by the nucleic acid tethers (**1**): (A) Absorption spectra of different concentrations of pNIPAM in the presence of a constant concentration of (**1**), corresponding to 0.5×10^{-6} M: (a) 0, (b) 1.25×10^{-5} M, (c) 2.5×10^{-5} M, (d) 5×10^{-5} M, (e) 7.5×10^{-5} M, (f) 1×10^{-4} M, (g) 1.25×10^{-4} M, (h) 1.5×10^{-4} M, (i) 1.75×10^{-4} M, (j) 2×10^{-4} M. (B) Calibration curve corresponding to the absorbance ratio $A_{\lambda 200}/A_{\lambda 255}$ as a function of pNIPAM/DNA ratio. Square marked with an arrow corresponded to the $A_{\lambda 200}/A_{\lambda 255}$ value of the synthesized (**1**)-modified pNIPAM copolymer implying a 1:168 ratio of (**1**) *vs.* NIPAM units.

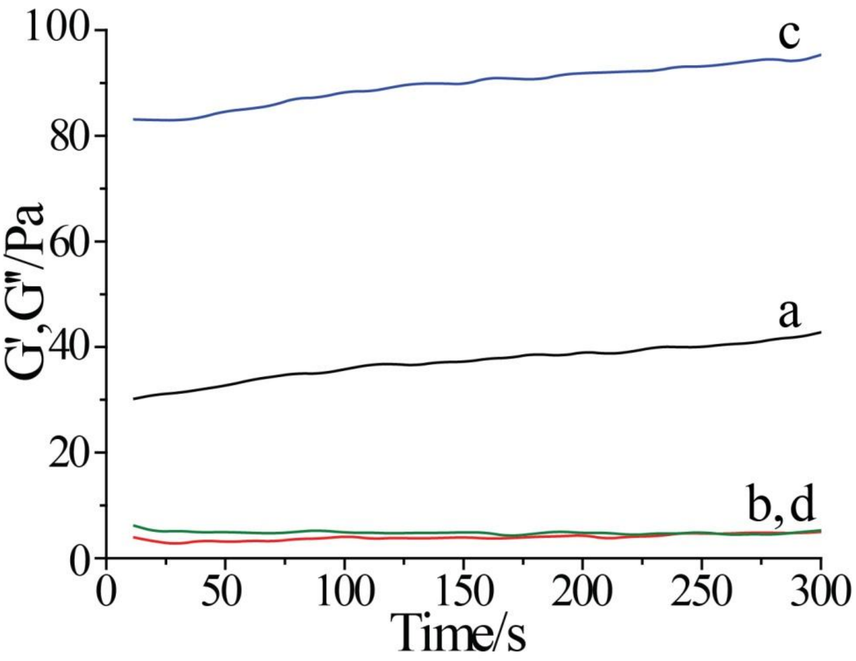

Figure S2. Rheology experiments characterizing the hydrogel produced according to Figure 2(A). Curve (a) and curve (b) show the storage modulus (G’) and the loss modulus (G”) of the G-quadruplex-crosslinked poly-N-isopropylacrylamide hydrogel. Curve (c) and (d) show the storage modulus (G’) and loss modulus (G”) of the hemin-G-quadruplex-crosslinked (1)-acrylamide/pNIPAM hydrogel/polyaniline hybrid (at pH 7.0).

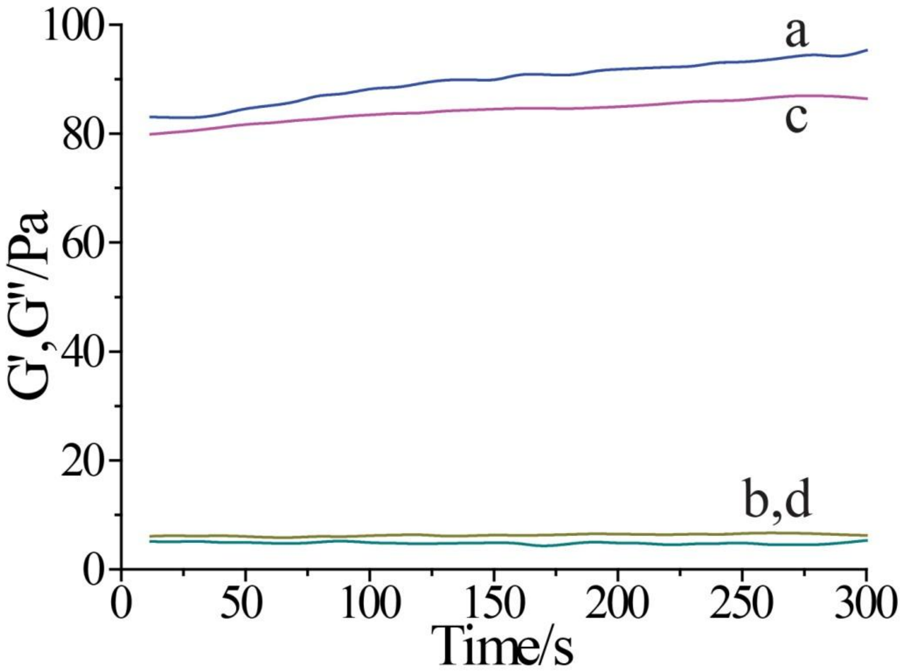

Figure S3. Rheology experiments characterizing the hemin-G-quadruplex-crosslinked (**1**)-acrylamide/pNIPAM hydrogel/polyaniline hybrid at different pH values. Curve (a) and (b) show the storage modulus (G’) and loss modulus (G”) of the hemin-G-quadruplex-crosslinked (**1**)-acrylamide/pNIPAM hydrogel/polyaniline hybrid at pH 7.0, respectively. Curve (c) and (d) show the storage modulus (G’) and loss modulus (G”) of the hemin-G-quadruplex-crosslinked (**1**)-acrylamide/pNIPAM hydrogel/ polyaniline hybrid at pH 3.0, respectively.

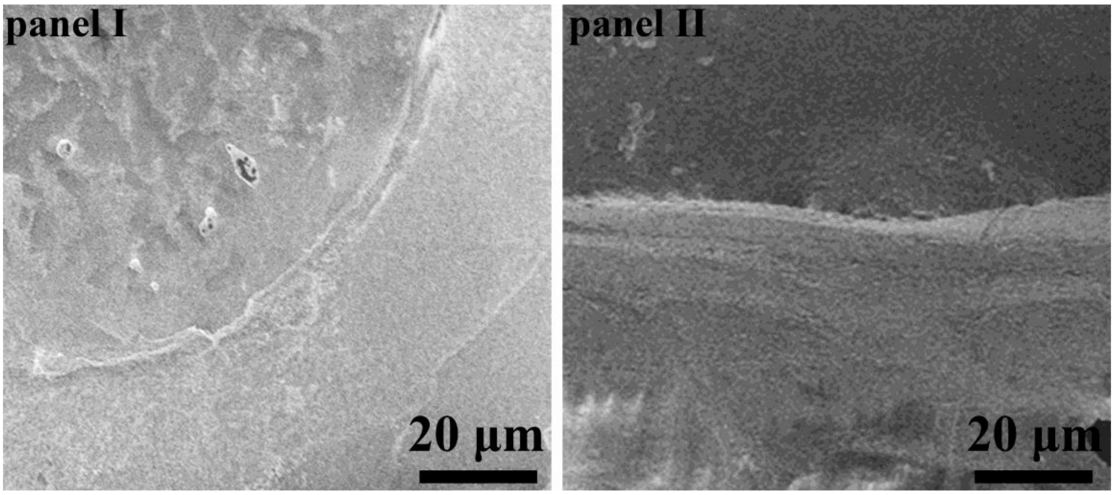

Figure S4. SEM images corresponding to the upper surface (panel I) and the cross section (Panel II) of the hemin-G-quadruplex-crosslinked (**1**)-acrylamide/pNIPAM hydrogel/polyaniline hybrid.
